# Novel Trivalent Vectored Vaccine for Control of Myxomatosis and Disease Caused by Classical and a New Genotype of Rabbit Haemorrhagic Disease Virus

**DOI:** 10.3390/vaccines8030441

**Published:** 2020-08-05

**Authors:** Sylvia Reemers, Leon Peeters, Joyce van Schijndel, Beth Bruton, David Sutton, Leo van der Waart, Saskia van de Zande

**Affiliations:** 1 Companion Animals R&D, MSD Animal Health, 5831 AN Boxmeer, The Netherlands; leon.peeters5@merck.com (L.P.); joyce.schijndel@merck.com (J.v.S.); beth.bruton@merck.com (B.B.); leo.vanderwaart@merck.com (L.v.d.W.); saskia.vandezande@merck.com (S.v.d.Z.); 2 Global Marketing Companion Animals, MSD Animal Health, Milton Keynes MK7 7AJ, UK; david.sutton@merck.com

**Keywords:** RHDV, myxomatosis, rabbit vaccine

## Abstract

Myxoma virus (MV) and rabbit haemorrhagic disease virus (RHDV) are the major causes of lethal viral diseases in the European rabbit. In 2010, a new RHDV genotype (RHDV2) emerged in the field that had limited cross-protection with the classical RHDV (RHDV1). For optimal protection of rabbits and preventing spread of disease, a vaccine providing protection against all three key viruses would be ideal. Therefore, a novel trivalent myxoma vectored RHDV vaccine (Nobivac Myxo-RHD PLUS) was developed similar to the existing bivalent myxoma vectored RHDV vaccine Nobivac Myxo-RHD. The new vaccine contains the Myxo-RHDV1 strain already included in Nobivac Myxo-RHD and a similarly produced Myxo-RHDV2 strain. This paper describes several key safety and efficacy studies conducted for European licensing purposes. Nobivac Myxo-RHD PLUS showed to be safe for use in rabbits from five weeks of age onwards, including pregnant rabbits, and did not spread from vaccinated rabbits to in-contact controls. Furthermore, protection to RHDV1 and RHDV2 was demonstrated by challenge, while the serological response to MV was similar to that after vaccination with Nobivac Myxo-RHD. Therefore, routine vaccination with Nobivac Myxo-RHD PLUS can prevent the kept rabbit population from these major viral diseases.

## 1. Introduction

Two major viral diseases affecting the European rabbit are myxomatosis and rabbit haemorrhagic disease (RHD). 

Myxomatosis is caused by myxoma virus, a member of the poxvirus family, genus *Leporipoxvirus*, and originated in the New World. While myxoma virus only causes mild disease in its natural host, the American rabbit, it causes serious disease and mortality in the European rabbit. However, it was not until the 1950s after being deliberately released in Australia and France that the virus became endemic [1]. The virus is primarily spread by blood feeding arthropod vectors such as fleas and mosquitos, although direct transmission or transmission via fomites has also been described [2,3,4]. After skin inoculation the virus replicates at the inoculation site in MHCII+ cells and spreads within leukocytes to the lymph nodes within 24 h. There it further replicates to high titres and disseminates via infected leukocytes to various organs [3]. Major sites of pathology are lymphoid tissues and the skin, resulting in typical clinical signs of oedematous swelling of eyelids, face, ears and the ano-genital area and serous nasal and conjunctival discharge [1,2]. Besides the nodular form of myxomatosis, there is also an amyxomatous form which is clinically milder (predominantly respiratory with fewer and smaller skin lesions) and generally nonlethal [2]. During the various outbreaks, attenuated myxoma virus strains have emerged in the field, and also the wild rabbit population experienced selection for resistance to myxomatosis. The emerging attenuated field strains can still be highly virulent (depending on the degree of virulence), but infected rabbits survive for longer in an infectious state, while resistant wild rabbits have reduced disease severity and higher survival rates [3]. With the wild rabbits acting as a reservoir of the disease the limited genetic resistance in domestic rabbits puts them at risk for myxomatosis. 

RHD is caused by RHD virus (RHDV), a member of the genus *Lagovirus* of the Caliciviridae family. RHDV transmission can be direct, via oral, nasal, conjunctival or parenteral routes or indirectly via fomites or insect vectors [5]. RHDV spreads throughout the entire body with liver, lung and spleen as the primary target tissues resulting in liver cell loss, splenomegaly, haemorrhages and T and B cell depletion accounting for the fatal progression of the disease [5]. RHDV was first reported in 1984 in China and appeared for the first time in Europe in 1986 [5]. In 2010, a completely new genotype referred to as RHDV2 or RHDVb appeared first in France and then spread across Europe [6]. Following the new proposed nomenclature [7], RHD viruses are currently divided into two genotypes: RHDV or classical RHDV (hereafter RHDV1) and RHDVa (a variant of RHDV) belong to genotype GI.1, while RHDV2 or RHDVb (hereafter RHDV2) belong to genotype GI.2. RHDV1 has a mortality rate of 70–100% in 36–96 h post infection. RHDV2 was initially reported to be less virulent than RHDV1 with a longer duration of disease and lower mortality rates. However, the pathogenicity of RHDV2 has increased over time which has resulted in a highly variable mortality between RHDV2 strains [6,8]. Furthermore, young rabbits and hare species are not resistant to disease caused by RHDV2 infection, unlike for RHDV1 [5,9,10]. Nowadays, RHDV outbreaks occur on almost all continents, being endemic in most parts of Europe, Asia and parts of Africa, Australia and New Zealand. Currently RHDV2 seems to be replacing RHDV1 in the field, and more pathogenic RHDV2 strains with mortality rates similar to RHDV1 have emerged and are becoming prevalent in the field [6].

The most effective method of preventing disease due to myxoma virus and RHDV and limiting outbreaks is vaccination. Over time various vaccines have been developed to protect against myxomatosis and RHD. Myxoma virus vaccines are live vaccines based on attenuated myxoma virus strains or the related Shope fibroma virus [1,11]. RHDV vaccines are mainly adjuvanted, inactivated virus vaccines containing virus derived from liver of infected rabbits, as RHDV does not grow in cell culture [12]. These vaccines provide protection against diseasecaused by either RHDV1 (hereafter RHD1), RHDV2 (hereafter RHD2) or both genotypes. There is only one vaccine licensed that provides protection against both myxomatosis and RHD1. This is the live recombinant attenuated myxoma virus vectored RHDV1 vaccine Nobivac Myxo-RHD [13]. However, there was no vaccine available that provides protection against myxomatosis, RHD1 and RHD2.

Therefore, a vaccine was developed that would provide protection against myxomatosis and both genotypes of RHDV. This vaccine was based on the existing live recombinant attenuated myxoma virus vectored RHDV1 vaccine, which already has an extensive proven efficacy and safety from years of use in the field. Here, we describe a number of safety and efficacy studies, in both commercial and pet rabbits, with the novel trivalent recombinant attenuated myxoma virus vectored RHDV1 and RHDV2 vaccine Nobivac Myxo-RHD PLUS. This vaccine was licensed in the EU in 2019.

## 2. Materials and Methods 

### 2.1. Animals

Studies were performed in 5-week-old specific pathogen free (SPF) New Zealand White (NZW) rabbits or SPF Dutch Belted (DB) rabbits (a typical pet breed) and 9-week-old SPF NZW rabbits or pregnant adult SPF NZW rabbits. NZW rabbits were obtained from Envigo, Huntingdon, UK, and DB rabbits were obtained from Covance, Denver, PA, USA. The SPF status of the animals and screening of blood samples at day of vaccination confirmed all animals were seronegative for myxoma virus and rabbit haemorrhagic disease virus (RHDV).

### 2.2. Ethics Statement

These studies were performed in compliance with the Dutch Experiments on Animals Act 2014 with approval of the MSD Animal Health Ethics Review Committees and Dutch Central Authority for Scientific Procedures on Animals (licence number AVD221002016638, approved 1 November 2016) or in compliance with the UK Animals Scientific Procedures Act 1986 (license number 70/8716, approved in 2015). After RHDV challenge rabbits were continuously monitored up to 72 h or until all controls succumbed to be able to immediately euthanize rabbits that showed signs of acute malaise to reduce animal suffering.

### 2.3. Vaccine

A newly updated recombinant live vector vaccine was used in the studies: Nobivac Myxo-RHD PLUS (MSD Animal Health, Boxmeer, The Netherlands). This vaccine is a combination of two live recombinant myxoma-vectored RHD viruses which were constructed as described previously [13]. In short, the capsid protein (VP60) of either RHDV1 (a German isolate of classical RHDV) or RHDV2 (a Spanish isolate of RHDV2) was inserted into the MGF/M11L locus of a laboratory-attenuated strain of myxoma virus using standard laboratory methods for homologous recombination. In this manner two live recombinant myxo-vectored RHDV viruses were created that have the same myxoma virus vector but contain the VP60 gene of either RHDV1 (strain 009, named hereafter Myxo-RHD1) or RHDV2 (strain MK1899, named hereafter Myxo-RHD2). Vaccine material was produced in RK-13 cells and for each of the studies a vaccine dose specific for the type of study was used. Nobivac Myxo-RHD PLUS is a lyophilised vaccine that needs to be reconstituted with solvent before use.

### 2.4. Viruses

All the RHD viruses used for challenge and serology testing were obtained from liver homogenate of RHDV-infected rabbits and titrated in rabbits, as RHDV does not grow in cell culture. As a representative of RHDV1, the RHDV Ascot strain was used, which was obtained from the Veterinary Laboratories Agency, Weybridge, UK [13]. Due to the changing nature of RHDV2 two representatives were used. RHDV2 Spain (MSD Animal Health, Milton Keynes, UK) was obtained from an outbreak in Spain in 2012 and used as a representative of an early RHDV2 strain. RHDV2 Italy2 was kindly provided by Dr. Capucci of the Istituto Zooprofilattico Sperimentale della Lombardia e dell’Emilia Romagna, Brescia, Italy, and was obtained from an outbreak in the Abruzzo region in Italy in 2015 [8]. This strain was used as a representative of a more recent RHDV2 strain with increased pathogenicity.

The myxoma virus (MSD Animal Health, Milton Keynes, UK) used for serology was similar to that previously described [13].

### 2.5. Vaccine Safety Studies

The design of studies 1 and 2 was based on the European Pharmacopoeia criteria for myxomatosis vaccine (live) for rabbits [14] and for rabbit haemorrhagic disease vaccine (inactivated) [15]. The design of study 3 was based on the EU licensing requirements and in accordance with Ph.Eur. 5.2.6 Evaluation of safety of veterinary vaccines and immunosera [16].

#### 2.5.1. Study 1 and 2: Vaccine Safety and Absence of Shedding of Vaccine Virus

Seventeen 5-week-old SPF NZW rabbits (11 vaccinated rabbits and 6 control rabbits) were used in study 1. Eighteen 5-week-old SPF DB rabbits (12 vaccinated rabbits and 6 control rabbits) were used in study 2. In each study rabbits were randomly divided into 2 groups and control rabbits were co-housed with vaccinated rabbits in cages. 

At day 0 rabbits in the vaccine group were vaccinated subcutaneously (s.c.) with a 10-fold overdose (≥10^6.8^ focus forming units (FFU) per virus strain per rabbit) of Nobivac Myxo-RHD PLUS vaccine reconstituted in 0.5 mL Nobivac Solvent, while the controls were inoculated with 0.5 mL Nobivac Solvent (placebo) (MSD Animal Health, Boxmeer, The Netherlands). At 21 days post vaccination (d.p.v.) vaccinated rabbits were revaccinated with a single maximum dose (≥10^5.8^ focus forming units (FFU) per virus strain per rabbit) of Nobivac Myxo-RHD PLUS vaccine, while the controls were inoculated with Nobivac Solvent. Before each inoculation, the injection site was shaved and was kept free of fur by repeated shaving to allow better monitoring of local reactions. Clinical monitoring of local reactions occurred daily for 2 weeks after each vaccination. Body temperature was monitored in accordance with the guideline for myxomatosis vaccine indicated above using a transponder (Plexx B.V., Elst, The Netherlands). Blood was sampled at day 0, day 21 and day 35 from all rabbits. At the end of the study (day 35) the injection sites were macroscopically examined for local reactions and tissue samples were taken from both injection sites of all vaccinates and 2 randomly selected controls.

#### 2.5.2. Study 3: Vaccine Safety in Pregnant Rabbits

Thirty proven mated SPF NZW rabbits were randomly divided into 4 groups and housed individually. Groups 1 and 2 comprised 10 rabbits each, and groups 3 and 4 comprised 5 rabbits each and served as unvaccinated controls. Groups 1 and 3 were used to evaluate vaccine safety in the first term of pregnancy and groups 2 and 4 for the second term of pregnancy. 

At day 0 of the study (day 7 after mating), each rabbit in group 1 was vaccinated s.c. with a maximum single dose (≥10^5.8^ FFU per virus strain per rabbit) of Nobivac Myxo-RHD PLUS reconstituted in 0.5 mL Nobivac Solvent and each rabbit of group 3 with 0.5 mL of Nobivac Solvent (placebo). At day 14 (day 21 after mating), each rabbit in group 2 was vaccinated s.c. with a maximum single dose of Nobivac Myxo-RHD PLUS reconstituted in 0.5 mL Nobivac Solvent and each rabbit of group 4 with 0.5 mL of Nobivac Solvent (placebo). Clinical monitoring, including local reactions, occurred daily until the end of study, and body temperature was monitored in accordance with the guideline for evaluation of safety of veterinary vaccines and immunosera indicated above using a transponder. Blood was sampled at day 0 and at parturition date + 1 day (end of study) of the adult rabbits. Following parturition, the numbers of kittens and live kittens and relative size of kittens were recorded.

### 2.6. Vaccine Efficacy Studies

For the design of studies 4 and 5 the European Pharmacopoeia criteria for rabbit haemorrhagic disease vaccine (inactivated) [15] was used as a guideline since no applicable monograph is available for live RHD vaccines.

#### 2.6.1. Study 4: Vaccine Efficacy to RHDV1 and RHDV2 Infection in SPF NZW Rabbits

Sixty SPF NZW rabbits were randomly divided into 8 groups, and each vaccinated group was floor housed together with a control group. Groups 1, 3, 5 and 7 comprised 10 rabbits each, and groups 2, 4, 6 and 8 comprised 5 rabbits each and served as unvaccinated controls. Groups 3 and 4 comprised 9-week-old rabbits and all other groups comprised 5-week-old rabbits at time of vaccination. Groups 3 and 4 comprised older animals because susceptibility to classical RHDV is age dependent. To evaluate vaccine efficacy in 5-week-old animals and an onset of immunity of 3 weeks, both 5- and 9-week-old rabbits had to be used (since animals of 8 weeks of age are unlikely to be susceptible). 

Rabbits in groups 1, 3, 5 and 7 were vaccinated s.c. with a single minimum antigen dose (≤10^3.0^ FFU per virus strain per rabbit) of Nobivac Myxo-RHD PLUS vaccine reconstituted in 0.5 mL Nobivac Solvent, while the controls were inoculated with 0.5 mL Nobivac Solvent (placebo). At 3 weeks post vaccination rabbits were challenged with RHDV: groups 3 and 4 with RHDV1 Ascot, groups 5 and 6 with RHDV2 Spain and groups 7 and 8 with RHDV2 Italy2. At 7 weeks post vaccination rabbits in groups 1 and 2 were challenged with RHDV1 Ascot. Monitoring of clinical signs of RHDV infection occurred for 14 days after challenge. Blood was sampled from all rabbits before vaccination and before challenge.

#### 2.6.2. Study 5: Vaccine Efficacy to RHDV1 and RHDV2 Infection in Pet Rabbits

Forty-five SPF DB rabbits were randomly divided into 6 groups and each vaccinated group was floor housed together with a control group. Groups 1, 3 and 5 comprised 10 rabbits each, and groups 2, 4 and 6 comprised 5 rabbits each and served as unvaccinated controls. Groups 3 and 4 comprised 9-week-old rabbits, and all other groups comprised 5-week-old rabbits at time of vaccination. Groups 3 and 4 comprised older animals because susceptibility to classical RHDV is age dependent, as described above in Section 2.6.1. 

Rabbits in groups 1, 3 and 5 were vaccinated s.c. with a single minimum antigen dose (≤10^3.0^ FFU per virus strain per rabbit) of Nobivac Myxo-RHD PLUS vaccine reconstituted in 0.5 mL Nobivac Solvent, while the controls were inoculated with 0.5 mL Nobivac Solvent (placebo). At 3 weeks post vaccination rabbits were challenged with RHDV: groups 3 and 4 with RHDV1 Ascot and groups 5 and 6 with RHDV2 Italy2. At 7 weeks post vaccination rabbits in groups 1 and 2 were challenged with RHDV1 Ascot. Monitoring of clinical signs of RHDV infection occurred for 14 days after challenge. Blood was sampled from all rabbits before vaccination and before challenge.

#### 2.6.3. RHDV Challenge

After optimizing the challenge model in negative control rabbits in order to induce 100% mortality, the following dilutions were used in the studies. For challenge material a 1/50 (for RHDV1 Ascot) or 1/100 (for both RHDV2 strains) dilution of 40% *w*/*v* liver homogenate from rabbits infected with one of the challenge strains was prepared. For challenge infection, each rabbit received 100 µL of this material via the ocular route (50 µL in each eye), 100 µL intranasally (50 µL in each nostril) and 200 µL orally.

### 2.7. Collection and Storage of Samples

Collected blood samples were transported to the laboratory for serum separation and stored at ≤−15 °C until testing. Tissue samples of injection sites stored in buffered formalin (Sigma-Aldrich Chemie N.V., Zwijndracht, The Netherlands) were transported to the laboratory for histological analysis and stored at ambient temperature until testing.

### 2.8. Serology

Serum was analysed for antibodies to myxoma virus, RHDV1 and RHDV2. Antibodies to myxoma virus were measured using an immunofluorescent test as previously described [13]. A titre <6 (log_2_) was regarded as negative. Antibodies to RHDV were measured using a haemagglutination inhibition (HI) assay as previously described [12,13], as no specific antibodies to RHDV2 were available to set up an ELISA at the start of the first study and to be able to compare RHDV1 antibody responses between Nobivac Myxo-RHD PLUS and its predecessor Nobivac Myxo-RHD (MSD Animal Health, Boxmeer, The Netherlands). For RHDV1, human O-type blood (Sanquin, Nijmegen, The Netherlands) was used, and for RHDV2, human B-type blood (Sanquin, Nijmegen, The Netherlands) was used. A titre <1 (log_2_) was regarded as negative. 

### 2.9. Macroscopic Examination and Histological Analysis of Tissue of the Injection Site 

At the end of studies 1 and 2, after necropsy of the rabbits, the injection sites of all vaccinated rabbits and of two randomly selected control rabbits were macroscopically and histologically analysed. Macroscopic analysis included presence, shape and colour of a local reaction, presence of vaccine residues, oedema, consistency of the tissue and abscess formation on the injection site. Tissue samples taken from these injection sites were fixed in buffered formalin, processed and embedded in paraffin wax (Fisher Scientific, Landsmeer, The Netherlands). Subsequent processing, staining with H&E (Fisher Scientific, Landsmeer, The Netherlands) and analysis of the samples for abnormalities by a pathologist was performed.

### 2.10. Clinical Observations

For studies 1 and 2 clinical monitoring and gross examination of the injection sites for local reactions was conducted by trained personnel who observed the animals at day −1, 0, 4 h, 7, 8, 10, 13/14, 15/16, 17/18, 21, 21 + 4 h and daily from day 22 until day 35. Observations included swelling of eyelids, nose, anus, mouth, external genitalia and ear base, ocular discharge, abnormal breathing, reduced appetite and abnormal attitude. Body temperature was measured at the same time of day at day −3, −2, −1, 0, 4 h, 1, 2, 3, 4, 21, 21 + 4 h, 22, 23, 24 and 25.

For study 3, clinical monitoring was conducted by trained personnel who observed the animals daily until the end of the study. Clinical observation was performed visually to reduce stress to the animal which may affect the course of pregnancy. Observations included ocular discharge, abnormal breathing, reduced appetite and abnormal attitude. Body temperature was measured at the same time of day at day −3, −2, −1 and 0 for all animals, at 4 h, day 1, 2, 3 and 4 for animals in groups 1 and 3 and at 14, 14 + 4 h, 15, 16, 17 and 18 for animals in groups 2 and 4.

For studies 4 and 5, clinical monitoring was conducted by trained personnel who observed the animals every two hours from 1 day up to 72 h post challenge or until all control rabbits were euthanized due to clinical signs caused by RHDV challenge, after which clinical observation continued once daily until the end of the study. Observations included abnormal breathing, abnormal attitude, position of the ears and mobility.

### 2.11. Monitoring of Gestation and Offspring

For study 3, all pregnant rabbits were monitored twice a day starting 5 days before the expected delivery date. Following parturition, the offspring of all rabbits were observed until 1 day after parturition. Observations included duration of gestation, number of does aborting, number of kittens born and, after parturition, the number of live kittens and the relative size of kittens (normal/abnormal). In addition, any deformities in the kittens were recorded.

### 2.12. Data and Statistical Analysis

To access vaccine safety, clinical signs (body temperature) between vaccinated and control rabbits were compared based on a repeated measures ANOVA model with the baseline temperature (on day 0 before vaccination) as a covariate in the model. A difference of *p* < 0.05 is considered significant. As baseline, the body temperature at day 0 per rabbit was used for analysis. In addition, vaccine virus spread to control rabbits housed together with vaccinated rabbits was analysed using a RHDV HI assay. Spreading is considered to have occurred when RHDV1 and RHDV2 HI titres are >4 (log_2_) in control rabbits.

To access vaccine safety during pregnancy, clinical signs (body temperature) were compared based on a repeated measures ANOVA model with the baseline temperature (on day 0 before vaccination) as a covariate in the model. A difference of *p* < 0.05 is considered significant. Descriptive statistics was used on gestation period, number of does aborting, litter size and number of still births. Results were compared between vaccinated and control rabbits inoculated in the same term of pregnancy.

To access vaccine efficacy against RHDV1 and RHDV2, the mortality data was summarized descriptively using frequency tables, mean, standard deviations and graphs where applicable. The Fisher’s exact test was used to compare the proportion of rabbits that survived the challenge, at 5% level of significance.

## 3. Results

### 3.1. Vaccine Safety Studies

#### 3.1.1. Vaccine Safety and Absence of Shedding of Vaccine Virus (Studies 1 and 2)

During clinical monitoring no clinical signs of myxomatosis were observed in any of the rabbits in either safety study (data not shown). Small, transient swellings up to 1.5 cm diameter and lasting up to 7 days were seen at the injection site in many cases following vaccination. These could be slightly larger (up to 2 cm diameter) and more persistent (up to 9 days) following vaccination at 10× overdose. Thickened skin could also be palpated at the majority of injection sites for up to 10 days. Furthermore, following overdose vaccination, a slight swelling of the local lymph node could be palpated for 1 day (Supplementary data Table S1 contains an overview of all observed clinical signs and local reactions).

Clinical examination, macroscopic analysis and histological analysis of the injection sites from animals in both studies indicated no abnormal reactions to vaccination other than injections site reactions within the normal limits upon subcutaneous injection (Supplementary Materials Table S2 and S3).

No significant difference in body temperature between vaccinated and control NZW rabbits was observed (Table 1). In vaccinated DB rabbits a maximum and mean increase in body temperature of respectively 0.8 °C and 0.4 °C was observed after the 10x overdose vaccination (Table 2). This was respectively 0.3 °C and 0.1 °C in control DB rabbits. There was evidence of a significant mean rise of 0.2 °C in the vaccinates (*p* = 0.0154) compared to the controls. This observed increase in body temperature is considered not biologically relevant and in accordance with the Ph.Eur. monograph for live myxomatosis vaccines. After repeated vaccination, the observed maximum increase was respectively 0.3 °C and 0.2 °C in the vaccinates and controls and no significant difference between vaccinates and controls was observed (*p* = 0.2040).

Serological data showed that all rabbits were seronegative for myxoma virus and RHDV at the start of the study. After vaccination, all vaccinated rabbits were seropositive for both myxoma virus and RHDV. With regard to virus shedding, none of the in-contact control rabbits that were housed together with the vaccinated rabbits became seropositive to either myxoma virus or RHDV (Supplementary Materials Table S4).

#### 3.1.2. Vaccine Safety during Pregnancy (Study 3)

No clinical signs of myxomatosis were observed in any of the rabbits during this study. In addition, no significant difference in body temperature was observed between vaccinated and control animals in both terms of the pregnancy (Table 3 and Table 4). 

Analysis of the gestation length and litter size revealed no differences between vaccinates and controls (Table 5). Furthermore, there were no abortions, no deformities and normal sized progeny in all groups. The offspring of two rabbits vaccinated in the second term of pregnancy included one dead kitten each. Post mortem examination demonstrated that both kittens were stillborn and that the death of both kittens was likely caused by dystocia (which was not seen because the parturition process of the does was not observed) and was, therefore, not related to the vaccination of the does. Three rabbits were not pregnant. One rabbit was an unvaccinated control for the first term of pregnancy group and was euthanized at study day 12 due to disease. The other two rabbits were vaccinates of the first and second term of pregnancy groups, which showed no signs of nesting behaviour and were euthanized for post mortem examination at 36 days after mating. No abnormalities were found in these two rabbits, and normal follicular development of the ovaries was observed. This indicates that treatment within this study was unlikely to be the cause of these rabbits not being pregnant, but this is instead likely to be related to the overall 90% pregnancy success rate of the supplier.

### 3.2. Vaccine Efficacy Studies

#### Vaccine Efficacy to Classical and a New Genotype of RHDV Challenge (Study 4 and 5)

Susceptibility to RHDV1 is age dependent while susceptibility to RHDV2 is not. To show an onset of immunity of 3 weeks for RHDV1 and susceptibility to vaccination at 5 weeks, rabbits were therefore vaccinated at two different ages. One group (group 3) was vaccinated at 9 weeks of age and challenged with RHDV1 3 weeks after vaccination, and another group (group 1) was vaccinated at 5 weeks of age and challenged with RHDV1 7 weeks after vaccination. By challenging at 12 rather than 8 weeks of age ensures the animal was susceptible to RHDV1 infection.

All rabbits were seronegative for myxoma virus and RHDV at the start of the study. All NZW and DB control rabbits remained seronegative for myxoma virus and RHDV until day of challenge. All, except three, vaccinated NZW rabbits were seropositive for myxoma virus and RHDV after vaccination. The exceptions were one rabbit from group 1 (RHDV1 challenge) and two rabbits from group 7 (RHDV2 Italy2 challenge). These three rabbits were seronegative to all three vaccine components (myxoma virus, RHDV1 and RHDV2), and therefore, it was concluded that they had most likely not been correctly vaccinated. All vaccinated DB rabbits were seropositive for myxoma virus and RHDV after vaccination (Table 6 and Table 7).

After RHDV challenges, all NZW and DB controls showed severe clinical signs indicative of RHDV infection and died within 120 h after RHDV challenge, independent of the challenge strain. All vaccinated NZW rabbits showed no clinical signs of RHDV infection and survived the challenge, except the three rabbits that showed no serological response. These three rabbits showed similar clinical signs of RHDV infection as the unvaccinated controls and died within 120 h after RHDV challenge. All vaccinated DB rabbits showed no clinical signs of RHDV infection and survived the challenge. Statistical analysis showed a significant difference in mortality after RHDV challenge between vaccinated and control rabbits within the same challenge group for all challenge groups for both NZW and DB rabbits (Table 8 and Table 9).

## 4. Discussion

Two major viral diseases affecting the rabbit population are RHD and myxomatosis. Since 2010, a new RHDV genotype has emerged (RHDV2), which at first was not as pathogenic as classical RHDV (RHDV1) but was able to affect both kittens and adults. However, over time the pathogenicity of RHDV2 viruses increased matching that of RHDV1, and nowadays, RHDV2 is replacing RHDV1 in the field [6,8,10]. As cross-protection between RHDV1 and RHDV2 is shown to be only partial [6,17], full protection to disease caused by RHDV will only be provided by a vaccine containing both genotypes. There are various vaccines commercially available that protect against disease caused by either myxoma virus alone or disease caused by RHDV1 and/or RHDV2 and only one vaccine that protects against both myxomatosis and RHD1. However, there was no vaccine available that will protect against disease caused by myxoma virus, RHDV1 and RHDV2 with one vaccination. Furthermore, RHDV cannot be cultured in vivo, and therefore, standard RHDV vaccines are prepared from livers of infected rabbits, which is not a desirable method with regard to animal welfare. New vaccines providing protection against RHD should therefore preferably be produced using an in vitro culture method.

Here, we demonstrate a novel trivalent myxoma virus vectored RHDV vaccine providing protection against disease caused by myxoma virus and both genotypes of RHDV that is produced using in vitro culturing. For this purpose the commercial vaccine strain of Nobivac Myxo-RHD (Myxo-RHD1) was used, because it has an extensive proven safety profile and efficacy profile against myxomatosis and RHD1 from years of use in the field, provides protection for a full 12 months following one initial dose and can be produced using in vitro culture. The Myxo-RHDV1 strain is composed of an attenuated strain of myxoma virus in which the VP60 gene of RHDV1 is inserted using homologous recombination. As a consequence of this insertion, two myxoma virus genes playing a role in viral virulence (MGF and ML11) have been removed, resulting in further attenuation of the myxoma virus [18,19] and, in this case particularly, in an attenuated myxoma virus vectored RHDV1 vaccine strain [13]. To keep all the properties of the Nobivac Myxo-RHD vaccine with regard to vaccine efficacy against myxomatosis and RHD1 (both onset and duration of immunity and efficacy in young rabbits with maternally-derived antibodies to myxoma virus and RHDV) and vaccine safety (no vaccine virus dissemination or spread), the same approach was used to create the Myxo-RHDV2 vaccine strain. Myxo-RHDV2 has the same myxoma virus vector as Myxo-RHDV1, and the VP60 of an RHDV2 strain was inserted in the same manner at the same position. Furthermore, by using two separate myxoma virus vectored strains instead of incorporation of the VP60 genes of both RHDV1 and RHDV2 in one myxoma virus vector, there is no risk of formation of new recombinants. Due to the nature of construction, Myxo-RHDV1 and Myxo-RHDV2 cannot form a new Myxo-RHDV1-RHDV2 strain, whereas a Myxo-RHDV1-RHDV2 strain could have resulted in formation of two new Myxo-RHDV1 and Myxo-RHDV2 strains. Based on that, the construct would have been proven genetically unstable, which is not in line with the regulations for construction of a myxoma virus vaccine [14]. By combining Myxo-RHDV1 and Myxo-RHDV2 together in the Nobivac Myxo-RHD PLUS vaccine, all the desirable properties of Nobivac Myxo-RHD vaccine were conserved and protection against disease by RHDV2 was additionally added.

As there has been discussion as to whether safety and efficacy results in a laboratory breed rabbit (NZW) is representative for a pet breed rabbit, a vaccine safety study and vaccine efficacy study were done in both a laboratory breed and pet breed rabbit. There were no differences in results between NZW and DB rabbits in either the vaccine safety or efficacy study using Nobivac Myxo-RHD PLUS. From this we can conclude that for Nobivac Myxo-RHD PLUS both breeds are suitable to prove vaccine safety and efficacy.

In line with EU guidelines vaccine safety was assessed by vaccinating rabbits with a 10-fold overdose and revaccinating with a single maximum commercial dose 3 weeks later. Minor local reactions (which were visible because the injection sites were kept free of fur by repeated shaving) and a minor increase in body temperature were observed in vaccinated rabbits. However, these observations were in line with vaccine safety regulations as indicated by the Ph.Eur. monograph for live myxomatosis vaccines [14]. Furthermore, the frequency of serious adverse reactions to vaccination in companion animals is extremely low and protection provided by vaccination far outweighs these reactions [20,21,22]. No spread of the vaccine strains from vaccinated rabbits to unvaccinated rabbits was observed; this is the same as has been previously shown for Nobivac Myxo-RHD. Spread of the vaccine virus via lymphocytes in the blood and ultimately via biting arthropods (main route of transmission) was based on the assessment for Nobivac Myxo-RHD vaccine and not repeated for Nobivac Myxo-RHD PLUS, as both vaccines contain the same myxoma virus vector. In this myxoma virus vector the M11L gene has been deleted, and consequently, the blood of vaccinated rabbits does not contain the vaccine strains and cannot, therefore, be spread via fleas or mosquitoes [13,18].

To determine vaccine safety during pregnancy, in line with EU guidelines, pregnant rabbits were vaccinated with a maximum commercial dose during either early or late gestation. Vaccination did not affect gestation length or litter size, nor did vaccination result in any signs of disease, abortions, deformities or differences in the size of the progeny. Two kittens were stillborn, but this was likely due to dystocia and unrelated to vaccination. In addition, three rabbits, of which one was an unvaccinated control rabbit, proved not to be pregnant. The other two rabbits were vaccinated in the first or second term of pregnancy, respectively. However, after post mortem examination no abnormalities and normal follicular development of the ovaries were found, indicating the lack of pregnancy is unlikely related to vaccination and more likely related to the overall 90% pregnancy success rate of the supplier. From these data we can conclude that the results are in line with EU guidelines for the evaluation of safety of veterinary vaccines and immunosera [16], and therefore, Nobivac Myxo-RHD PLUS is considered safe for use in pregnant rabbits.

In line with EU guidelines, vaccine efficacy was assessed by vaccinating rabbits with a single minimum commercial dose and challenging with RHDV 3 weeks later. Due to the variable pathogenicity of RHDV2 strains [6,8,10], rabbits were challenged with an early RHDV2 strain (RHDV2 Spain) or a recent RHDV2 strain (RHDV2 Italy2). As RHDV1 strains do not show this amount of variability in pathogenicity, one RHDV1 strain was used for challenge. The analysis of both studies showed 0% protection to RHDV challenge in the controls independent of the RHDV challenge strain used. In the study of DB rabbits, 100% protection to RHDV challenge was observed in the vaccinates independent of the RHDV challenge strain used. In the study of NZW rabbits, vaccinates showed 90%, 100%, 100% and 80% protection to respectively RHDV1 Ascot for group 1 and 3, RHDV2 Spain and RHDV2 Italy2. The three vaccinated rabbits that did not survive challenge were seronegative for myxoma virus, RHDV1 and RHDV2, indicating they had probably not been vaccinated properly. This is also corroborated by the clinical signs typical for RHDV infection in these animals and the time of death (within 120 h after RHDV infection), which were similar to the unvaccinated controls. If these three rabbits are excluded, this results in 100% protection against RHDV challenge irrespective of the challenge strain. If these three rabbits are included there is still a significant difference in mortality between vaccinates and unvaccinated controls. Efficacy to RHDV1 challenge was comparable between Nobivac Myxo-RHD and Nobivac Myxo-RHD PLUS as expected based upon inclusion of strain 009 in both vaccines. However, antibody titres to RHDV1 may appear higher for Nobivac Myxo-RHD, as shown by Spibey et al., 2012 [13], compared to the data present here for Nobivac Myxo-RHD PLUS. This difference results from a difference in time point of blood sampling after vaccination. While Spibey et al., 2012 [13], sampled blood 11 weeks post vaccination, in our study blood was sampled 3 weeks post vaccination for all groups. RHDV1 and RHDV2 antibody responses reach their peak at approximately 13 weeks post vaccination. Therefore, antibody titres to RHDV1 as shown by Spibey et al., 2012 [13], were higher compared to our data. However, when antibody titres to RHDV1 were compared at the same time point post vaccination, antibody titres were comparable between Nobivac Myxo-RHD and Nobivac Myxo-RHD PLUS (based on data vaccine licensing procedures, data not shown). From these data we can conclude that the results are in line with EU guidelines for rabbit haemorrhagic disease vaccine (inactivated) [15] and that vaccination with Nobivac Myxo-RHD PLUS provides efficacious protection against disease caused by RHDV1 and RHDV2. In line with the principles of the 3Rs for animal welfare and EU guidelines, vaccine efficacy against myxomatosis was based upon the available results for strain 009 included in Nobivac Myxo-RHD. Since both strain 009 and MK1899 are identical except for the inserted RHDV1 or RHDV2 VP60 gene and there is no expression of the RHDV VP60 (capsid) protein on the outside of the myxoma virus particle, the efficacy of the immune response against myxoma virus cannot be negatively affected by the addition of an another myxoma-RHDV vaccine strain based on the same backbone. This was confirmed using serology. Antibody titres to myxoma virus were comparable between Nobivac Myxo-RHD and Nobivac Myxo-RHD PLUS (based on data vaccine licensing procedures, data not shown).

## 5. Conclusions

In conclusion, these vaccine registration studies with a novel trivalent myxoma virus vectored RHDV vaccine (Nobivac Myxo-RHD PLUS) confirm that this vaccine is safe for use in rabbits from 5 weeks of age onwards, including pregnant rabbits. The studies also confirm that a single dose of the vaccine provides protection against disease caused by myxoma virus, RHDV1 and RHDV2 from 3 weeks until 12 months post vaccination (latter data not shown, based on license approval). As an additional welfare benefit, this vaccine is produced in vitro and not in live rabbits.

## Figures and Tables

**Table 1:** Average body temperature (°C) in New Zealand White (NZW) rabbits per day.

Treatment Group	-	Vaccination														
		10× Overdose									Repeated Dose					
	Day	−3	−2	−1	0	0 + 4 h	1	2	3	4	21	21 + 4 h	22	23	24	25
Controls	Mean	39.1	39.1	39.2	39.2	39.3	39.2	39.2	39.1	39.2	39.0	39.0	38.9	38.8	39.0	38.9
	Max	39.6	39.2	39.5	39.4	39.4	39.5	39.5	39.4	39.6	39.3	39.2	39.2	39.2	39.4	39.2
Vaccinates	Mean	39.2	39.1	39.2	39.2	39.2	39.2	39.1	39.4	39.2	38.8	39.1	38.9	38.8	38.9	38.7
	Max	39.9	39.5	39.6	39.6	39.6	39.5	39.5	39.8	39.5	39.1	39.4	39.5	39.2	39.5	39.0

**Table 2:** Average body temperature (°C) in in Dutch Belted (DB) rabbits per day.

Treatment Group	-	Vaccination														
		10× Overdose									Repeated Dose					
	Day	−3	−2	−1	0	0 + 4 h	1	2	3	4	21	21 + 4 h	22	23	24	25
Controls	Mean	39.2	39.0	39.0	39.1	39.2	39.0	38.9	39.0	39.0	38.9	38.7	38.9	38.8	38.7	38.9
	Max	39.8	39.5	39.5	39.5	39.6	39.5	39.3	39.5	39.6	39.1	39.0	39.4	39.0	38.8	39.3
Vaccinates	Mean	39.1	39.1	39.0	39.1	39.3	39.1	39.4	39.3	39.1	39.0	39.1	39.0	38.9	38.8	38.8
	Max	39.3	39.7	39.4	39.5	39.6	39.4	39.9	39.6	39.7	39.3	39.5	39.4	39.2	39.2	39.4

**Table 3:** Average body temperature (°C) in the first term of pregnancy in rabbits per day.

Treatment Group	Day	−3	−2	−1	0	0 + 4 h	1	2	3	4
Vaccinates	Mean	39.3	39.3	39.2	39.2	39.0	39.2	39.3	39.3	39.4
	Max	39.5	39.6	39.6	39.5	39.3	39.6	39.8	39.5	39.9
Controls	Mean	39.1	39.1	38.8	39.0	38.8	38.9	38.9	38.9	39.0
	Max	39.4	39.4	39.0	39.2	39.1	39.2	39.2	39.1	39.4

**Table 4:** Average body temperature (°C) in the second term of pregnancy in rabbits per day.

Treatment Group	Day	−3	−2	−1	0	14	14 + 4 h	15	16	17	18
Vaccinates	Mean	39.0	39.1	38.9	39.1	39.0	38.6	38.8	38.8	38.9	38.8
	Max	39.4	39.5	39.5	39.8	39.5	39.1	39.3	39.3	39.4	39.2
Controls	Mean	38.8	39.0	38.7	38.9	38.9	38.7	38.8	38.7	38.7	38.5
	Max	39.1	39.5	39.3	39.7	39.8	39.1	39.9	39.4	39.3	38.6

**Table 5:** Summary of gestation monitoring and offspring.

Pregnancy Term	-	Gestation Duration (Days)			Litter Size		
		Mean	Min	Max	Mean	Min	Max
First	Vaccinates	30.6	30	31	7.7	5	11
	Controls	31.0	31	31	6.5	5	9
Second	Vaccinates	30.8	30	32	7.7	6	11
	Controls	31.0	30	32	7.2	4	11

**Table 6:** Average antibody titre (log_2_) to classical rabbit haemorrhagic disease virus (RHDV1), the new RHDV genotype (RHDV2) and myxoma virus in NZW rabbits.

Treatment Group			Titre to RHDV1		Titre to RHDV2		Titre to Myxoma Virus	
Challenge Virus	-	Group	Day 0 or 28 ^ǂ^	Day 49	Day 0 or 28 ^ǂ^	Day 49	Day 0 or 28 ^ǂ^	Day 49
RHDV1 Ascot	Vaccinates	1	<1 *	3.3	<1	5.1	<6 ^#^	9.9
	Controls	2	<1	<1	<1	<1	<6	<6
RHDV1 Ascot	Vaccinates	3	<1	2.4	<1	4.5	<6	12.4
	Controls	4	<1	<1	<1	<1	<6	<6
RHDV2 Spain	Vaccinates	5	<1	3.0	<1	3.8	<6	12.4
	Controls	6	<1	<1	<1	<1	<6	<6
RHDV2 Italy2	Vaccinates	7	<1	2.4	<1	4.0	<6	11.2
	Controls	8	<1	<1	<1	<1	<6	<6

* For RHDV a titre <1 is considered negative; ^#^ for myxoma virus a titre <6 is considered negative; ^ǂ^ blood was sampled prior to vaccination for group 1 and 2 at day 0 and for group 3–8 at day 28.

**Table 7:** Average antibody titre (log_2_) to classical rabbit haemorrhagic disease virus (RHDV1), the new RHDV genotype (RHDV2) and myxoma virus in DB rabbits.

Treatment Group			Titre to RHDV1		Titre to RHDV2		Titre to Myxoma Virus	
Challenge Virus	-	Group	Day 0 or 29 ^ǂ^	Day 21 or 49 ^§^	Day 0 or 29 ^ǂ^	Day 21 or 49	Day 0 or 29 ^ǂ^	Day 21 or 49
RHDV1 Ascot	Vaccinates	1	<1 *	4.3	<1	6.9	<6 ^#^	9.4
	Controls	2	<1	<1	<1	<1	<6	<6
RHDV1 Ascot	Vaccinates	3	<1	4.5	<1	6.4	<6	9.2
	Controls	4	<1	<1	<1	<1	<6	<6
RHDV2 Italy2	Vaccinates	5	<1	3.9	<1	6.9	<6	10.9
	Controls	6	<1	<1	<1	<1	<6	<6

* For RHDV a titre <1 is considered negative; ^#^ for myxoma virus a titre <6 is considered negative; ^ǂ^ blood was sampled prior to vaccination for group 1, 2, 5 and 6 at day 0 and for group 3 and 4 at day 29; ^§^ blood was sampled prior to challenge for group 5 and 6 at day 21 and for group 1–4 at day 49.

**Table 8:** Summary of mortality after RHDV challenge in NZW rabbits.

Challenge Virus	-	Group (No. Rabbits)	Survival (%)	Death (%)	*p*-Value Vaccinates vs. Controls *
RHDV1 Ascot	Vaccinates	1 (10)	90	10	0.0020
	Controls	2 (5)	0	100	
RHDV1 Ascot	Vaccinates	3 (10)	100	0	0.0003
	Controls	4 (5)	0	100	
RHDV2 Spain	Vaccinates	5 (10)	100	0	0.0003
	Controls	6 (5)	0	100	
RHDV2 Italy2	Vaccinates	7 (10)	80	20	0.0070
	Controls	8 (5)	0	100	

* Statistical difference in mortality to RHDV challenge between vaccinated and control rabbits.

**Table 9:** Summary of mortality after RHDV challenge in DB rabbits.

Challenge Virus	-	Group (No. Rabbits)	Survival (%)	Death (%)	*p*-Value Vaccinates vs. Controls *
RHDV1 Ascot	Vaccinates	1 (10)	100	0	0.0003
	Controls	2 (5)	0	100	
RHDV1 Ascot	Vaccinates	3 (10)	100	0	0.0003
	Controls	4 (5)	0	100	
RHDV2 Italy2	Vaccinates	5 (10)	100	0	0.0003
	Controls	6 (5)	0	100	

* Statistical difference in mortality to RHDV challenge between vaccinated and control rabbits.

## Supplementary Materials

The following are available online at https://www.mdpi.com/2076-393X/8/3/441/s1, Table S1A: Observed clinical and local reactions in study 1 (specific pathogen free (SPF) rabbits), Table S1B: Observed clinical and local reactions in study 2 (DB rabbits), Table S2A: Macroscopic analysis injection sites, Table S2B: Macroscopic analysis injection sites in study 1 (SPF rabbits), Table S2C: Macroscopic analysis injection sites in study 2 (DB rabbits), Table S3A: Histological analysis of the injection sites in study 1 (SPF rabbits), Table S3B: Histological analysis of the injection sites in study 2 (DB rabbits), Table S4: Summary of antibody titres to RHDV1, RHDV2 and myxoma virus (S4A NZW rabbits, S4B DB rabbits).
