# Supplementary material for: Novel Trivalent Vectored Vaccine for Control of Myxomatosis and Disease Caused by Classical and a New Genotype of Rabbit Haemorrhagic Disease Virus
Source: Vaccines (Basel). 2020 Aug 5;8(3):441. doi: 10.3390/vaccines8030441 (PMC7565868; doi:10.3390/vaccines8030441)
Supplement: Supplementary file 1 [file vaccines-08-00441-s001.pdf]

Supplementary data Myxo-RHD PLUS manuscript

Table S1A. Observed clinical and local reactions in Study 1 (SPF rabbits)

Study day	Rabbit	Local reaction left injection site	Local reaction right injection site
Day 14	3.2 (v)	1 cm diameter	N.a.
	Other vaccinates	Thickened skin	N.a.
	All controls	No	N.a.
Day 15	3.2 (v)	1 cm diameter, enlarged lymphode 5 mm diameter	N.a.
	3.3., 3.5 (vaccinate representatives	5 mm diameter, slightly enlarged lymphode 3 mm diameter	N.a.
	3.1 (control representative)	No	N.a.
Day 16	3.2 (v)	1 x 0.5 cm	N.a.
	2.5, 2.6, 3.3. 3.5, 3.6, 4.1, 4.3 (v)	Thickened skin	N.a.
	Other vaccinates	No	N.a.
	All controls	No	N.a.
Day 18	3.2 (v)	1 x 0.5 cm	N.a.
	3.6, 4.1, 4.3 (v)	Thickened skin	N.a.
	Other vaccinates	No	N.a.
	All controls	No	N.a.
Day 21	3.2 (v)	1 x 0.5 cm	N.a.
	Other vaccinates	No	N.a.
	All controls	No	N.a.
Day 21 + 4 hrs	2.5 (v)	N.a.	Thickened skin
	Other vaccinates	N.a.	No
	All controls	N.a.	No
Day 22	3.2 (v)	0.5 cm in diameter	Thickened skin
	2.5, 4.1, 4.3 (v)	No	Thickened skin
	Other vaccinates	No	No
	All controls	No	No
Day 23	3.2 (v)	0.5 cm in diameter	0.7 x 1 cm
	2.5 (v)	No	0.5 x 1.5 cm
	2.6 (v)	No	0.5 cm in diameter
	4.1, 4.3 (v)	No	Thickened skin
	Other vaccinates	No	No
	All controls	No	No
Day 24	3.2 (v)	0.3 cm in diameter	0.5 x 1 cm
	2.5 (v)	No	0.5 x 1 cm
	2.6 (v)	No	0.3 cm in diameter
	2.8, 4.1 (v)	No	Thickened skin
	Other vaccinates	No	No
	All controls	No	No
Day 25	3.2 (v)	0.3 cm in diameter	0.5 x 1 cm
	2.5 (v)	No	0.5 cm in diameter
	2.8, 4.1 (v)	No	Thickened skin
	Other vaccinates	No	No
	All controls	No	No
Day 26	3.2 (v)	0.2 cm in diameter	0.5 x 1 cm
	2.5 (v)	No	0.5 cm in diameter
	2.8, 4.1 (v)	No	Thickened skin
	Other vaccinates	No	No
	All controls	No	No

(v): vaccinated rabbit
(c): unvaccinated control rabbit

Table S1A. Observed clinical and local reactions in Study 1 (SPF rabbits)

Study day	Rabbit	Local reaction left injection site	Local reaction right injection site
Day 27	3.2 (v)	0.2 cm in diameter	0.5 x 1 cm
	2.5 (v)	No	0.5 cm in diameter
	2.8, 4.1 (v)	No	Thickened skin
	Other vaccinates	No	No
	All controls	No	No
Day 28	3.2 (v)	0.2 cm in diameter	0.2 x 0.5 cm
	2.5 (v)	No	0.3 cm in diameter
	2.7 (c)	No	1 x 1.5 cm
	Other vaccinates	No	No
	All controls	No	No
Day 29	3.2 (v)	0.2 cm in diameter	0.2 cm in diameter
	2.7 (c)	No	1.5 cm in diameter
	Other vaccinates	No	No
	All controls	No	No
Day 30	3.2 (v)	0.2 cm in diameter	No
	2.7 (c)	No	1.5 cm in diameter
	Other vaccinates	No	No
	All controls	No	No
Day 31	3.2 (v)	0.2 cm in diameter	No
	2.7 (c)	No	1.5 cm in diameter
	Other vaccinates	No	No
	All controls	No	No
Day 32	3.2 (v)	0.2 cm in diameter	No
	2.3 (c)	No	small scrabs
	2.7 (c)	No	1.5 cm in diameter
	Other vaccinates	No	No
	All controls	No	No
Day 33	3.2 (v)	0.2 cm in diameter	No
	2.3 (c)	No	small scrabs
	2.7 (c)	No	1.5 cm in diameter
	Other vaccinates	No	No
	All controls	No	No
Day 34	3.2 (v)	0.2 cm in diameter	No
	2.3 (c)	No	small scrabs
	2.7 (c)	No	1.5 cm in diameter
	Other vaccinates	No	No
	All controls	No	No
Day 35	2.3 (c)	No	scabs
	2.7 (c)	No	1.5 cm in diameter
	Other vaccinates	No	No
	All controls	No	No

(v): vaccinated rabbit
(c): unvaccinated control rabbit

Table S1B. Observed clinical and local reactions in Study 2 (DB rabbits)

Study day	Rabbit	Local reaction left injection site	Local reaction right injection site
Day 7	2.3 (v)	0.5 x 1.5 cm diameter	N.a.
	Other vaccinates	No	N.a.
	All controls	No	N.a.
Day 8	1.1; 1.3; 2.5; 3.1; 4.1; 4.3; 4.4 (v)	Thickened skin	N.a.
	2.3 (v)	0.5 x 1.5 cm diameter	N.a.
	Other vaccinates	No	N.a.
	All controls	No	N.a.
Day 10	1.3; 2.2; 2.5; 2.6; 4.4 (v)	Thickened skin	N.a.
	1.1; 3.1 (v)	1 x 2 cm diameter	N.a.
	2.3 (v)	1 x 1.5 cm diameter	N.a.
	Other vaccinates	No	N.a.
	All controls	No	N.a.
Day 13	1.3; 2.2; 2.5; 2.6; 4.4 (v)	Thickened skin	N.a.
	1.1; 2.3 (v)	1 x 1.5 cm diameter	N.a.
	3.1 (v)	1 x 2 cm diameter	N.a.
	Other vaccinates	No	N.a.
	All controls	No	N.a.
Day 15	2.5; 2.6; 4.4 (v)	Thickened skin	N.a.
	1.1; 2.3 (v)	1 cm in diameter	N.a.
	3.1 (v)	1 x 1.5 cm diameter	N.a.
	Other vaccinates	No	N.a.
	All controls	No	N.a.
Day 17	1.1; 2.3; 2.5; 2.6; 4.4 (v)	Thickened skin	N.a.
	3.1 (v)	0.2 x 1 cm	N.a.
	Other vaccinates	No	N.a.
	All controls	No	N.a.
Day 23	1.3; 3.1 (v)	N.a.	0.5 cm in diameter
	Other vaccinates	N.a.	No
	All controls	N.a.	No
Day 24	1.3 (v)	N.a.	0.5 cm in diameter
	3.1 (v)	N.a.	1 cm in diameter
	Other vaccinates	N.a.	No
	All controls	N.a.	No
Day 25	1.3; 2.5 (v)	N.a.	0.5 cm in diameter
	3.1 (v)	N.a.	1 cm in diameter
	Other vaccinates	N.a.	No
	All controls	N.a.	No
Day 26	1.3; 2.5 (v)	N.a.	0.5 cm in diameter
	3.1 (v)	N.a.	1 cm in diameter
	Other vaccinates	N.a.	No
	All controls	N.a.	No
Day 27	1.3 (v)	N.a.	0.2 cm in diameter
	2.5; 3.1 (v)	N.a.	0.5 cm in diameter
	Other vaccinates	N.a.	No
	All controls	N.a.	No

(v): vaccinated rabbit
(c): unvaccinated control rabbit

Table S2A. Macroscopic analysis injection sites

MACROSCOPIC ANALYSIS AND SCORING OF LOCAL REACTIONS

*Aspects of the local reaction:
- 0: no local reaction present
- 1: homogenous area
- 2: irregular area

* Color of the local reaction: one of the option below will be chosen:
- 0: normal
- 1: pale (grey discoloration)
- 2: white color (possibly near vaccine-/test article residues)
- 3: redness
- 4: yellow discoloration
- 5: green discoloration
- 6: other (report explicitly)

* Presence of clear recognizable test article residues:
(possible adjuvant residues will be considered as test article residues)
- 0: no
- 1: minimal test article residues (<10% of the administered volume)
- 2: extensive vaccine/ test article residues (≥10% of the administered volume)
- 3: vaccine/ test article residues are spread over multiple separated sites

* presence of haemorrhages on the area of administration:
- 0: no
- 1: minimal haemorrhages (limited haemorrhages in the direct area of administration)
- 2: extensive haemorrhages (severe haemorrhages near and around the area of administration)
- 3: presence of clotted blood on and around the area of administration

* Oedema on the area of administration:
- 0: no
- 1: minimal oedema (limited oedema near the area of administration)
- 2: extensive oedema (severe oedema near and around the area of administration)

* Consistency of the tissue on the area of administration:
- 0: no changes, same consistency as surrounding tissue
- 1: softening
- 2: hardening

* Abscess formation on the area of administration:
- 0: absent
- 1: abscess formation (diameter smaller or equal to ±1cm)
- 2: abscess formation (diameter larger than ±1cm)
- 3: in case several abscesses also note the amount of abscesses

Table S2B. Macroscopic analysis injection sites Study 1 (SPF rabbits)

Group	Rabbit number	Size (hxwxd) (cm)	Aspects	Color	Vaccin/ test article residue	Heamorrhages	Oedeem	Consistency	Abscess
Vaccinates	2.1 R	-	0	0	0	0	0	0	0
	2.1 L	-	0	0	0	0	0	0	0
	2.2 R	-	0	0	0	0	0	0	0
	2.2 L	1.0x1.0x0.1	1	4	0	0	0	0	0
	2.5 R	1.0x1.0x0.1	1	4	0	0	0	0	0
	2.5 L	-	0	0	0	0	0	0	0
	2.6 R	-	0	0	0	0	0	0	0
	2.6 L	-	0	0	0	0	0	0	0
	2.8 R	-	0	0	0	0	0	0	0
	2.8 L	0.5x0.5x0.1	1	4	0	0	0	0	0
	3.2 R	-	0	0	0	0	0	0	0
	3.2 L	0.1x0.1x0.05	1	3	0	1	0	0	0
	3.3 R#	1.5x1.0x0.1	1	3	0	1	0	0	0
	3.3 L	-	0	0	0	0	0	0	0
	3.5 R	1.0x1.0x0.1	1	3/4	0	0	0	0	0
	3.5 L	-	0	0	0	0	0	0	0
	3.6 R	1.0x0.5x0.1	1	3	0	1	0	0	0
	3.6 L	1.0x1.0x0.1	1	4	0	0	0	0	0
	4.1 R	0.5x0.5x0.1	1	4	0	1	0	0	0
	4.1 L	-	0	0	0	0	0	0	0
	4.3 R	1.0x0.1x0.1	1	4	0	1	0	0	0
	4.3 L	-	0	0	0	0	0	0	0
Controls	2.3 R*	-	0	0	0	0	0	0	0
	2.3 L	-	0	0	0	0	0	0	0
	2.4 R	0.5x0.5x0.1	1	4	0	0	0	0	0
	2.4 L	1.0x1.0x0.1	1	4	0	0	0	0	0

R: right injection site (repeated dose)
L: left injection site (10x overdose)
* Scabs on the surface of the skin
# Hemorrhage is located on the location of the transponder

Table S2C. Macroscopic analysis injection sites Study 2 (DB rabbits)

Group	Rabbit number	Size (hxwxd) (cm)	Aspects	Color	Vaccin/ test article residue	Heamorrhages	Oedeem	Consistency	Abscess
Vaccinates	1.1 L	-	0	0	0	0	0	0	0
	1.1 R	-	0	0	0	0	0	0	0
	1.3 L	-	0	0	0	0	0	0	0
	1.3 R	-	0	0	0	0	0	0	0
	2.2 L	-	0	0	0	0	0	0	0
	2.2 R	-	0	0	0	0	0	0	0
	2.3 L	-	0	0	0	0	0	0	0
	2.3 R	-	0	0	0	0	0	0	0
	2.5 L	-	0	0	0	0	0	0	0
	2.5 R	-	0	0	0	0	0	0	0
	2.6 L	-	0	0	0	0	0	0	0
	2.6 R	-	0	0	0	0	0	0	0
	3.1 L	-	0	0	0	0	0	0	0
	3.1 R	-	0	0	0	0	0	0	0
	4.1 L	-	0	0	0	0	0	0	0
	4.1 R	-	0	0	0	0	0	0	0
	4.3 L	-	0	0	0	0	0	0	0
	4.3 R	-	0	0	0	0	0	0	0
	4.4 L	-	0	0	0	0	0	0	0
	4.4 R	-	0	0	0	0	0	0	0
	4.5 L	-	0	0	0	0	0	0	0
	4.5 R	-	0	0	0	0	0	0	0
Controls	1.2 L	-	0	0	0	0	0	0	0
	1.2 R	-	0	0	0	0	0	0	0
	2.1 L	-	0	0	0	0	0	0	0
	2.1 R	-	0	0	0	0	0	0	0

R: right injection site (repeated dose)
L: left injection site (10x overdose)

Table S3A. Histological analysis of the injection sites Study 1 (SPF rabbits)

Animal no.	Histological description injection site
2.1R	No abnormalities detected
2.1L	No abnormalities detected
2.2R	No abnormalities detected
2.2L	No abnormalities detected
2.3R	Ulceration of the epidermis with crust formation on the surface and associated with area of edema, haemorrhage and aggregation of hypertrophic fibroblasts spindle shaped with large nuceli/nucleoli.
2.3L	No abnormalities detected
2.4R	First slide: No abnormalities detected, but epidermis missing. Therefore extra slides made.<br>Extra slides (4x) : Epidermis present; no abnormalities detected
2.4L	First slide: No abnormalities detected, but epidermis missing. Therefore extra slides made.<br>Extra slides (3x): Epidermis present; no abnormalities detected
2.5R	No abnormalities detected
2.5L	First slide: Focally perivascular mononuclear cell aggregation in the dermis. Mild degeneration of (single) muscle fibers associated with area of edema. Subdermal small capillaries congested. But epidermis almost completely absent. Therefore extra slides made.<br>Extra slides (3x): Epidermis present; 2x no abnormalities detected, 1x degeneration of single muscle fiber associated with minimal mononuclear cell aggregation. Focally submuscular subcutaneous mononuclear cell aggregation with vacuolated macrophages (multinucleated)
2.6R	Focally degenerative and regenerative changes in single muscle fibers
2.6L	No abnormalities detected
2.8R	No abnormalities detected
2.8L	Focally perivascular mononuclear cell aggregation associated with proliferative changes of the endothelium
3.2R	No abnormalities detected
3.2L	Focally mild degeneration of (single) muscle fibers
3.3R	Focally minimal mononuclear (plasma cells) cell aggregation in the dermis. Subcutaneous minimal edema with presence of few hypertrophic fibroblasts and congestion of microcapillaries
3.3L	No abnormalities detected
3.5R	Mild perivascular and intramuscular mononuclear cell aggregation
3.5L	Minimal subdermal mononuclear cell aggregation associated with degenerative /regenerative changes of (single) muscle fibers
3.6R	Subcutaneous area with edema, hemorrhages, congested microcapillaries and hyperplastic fibroblasts. Degeneration of single muscle fibers
3.6L	Focally minimal mononuclear cell aggregation . area with subcutaneous edema.
4.1R	No abnormalities detected
4.1L	No abnormalities detected
4.3R	Subcutaneous mild mononuclear cell aggregation (adipose tissue). Dermal area with minimal haemorrhage. Focally perifollicular mononuclear cell aggregation
4.3L	Focally perivasculitis with mononuclear cell aggregation in the lamina muscularis. Minimal degeneration of (single) muscle fibers sometimes associated with minimal mononuclear cell aggregation

Vaccinates: 2.1, 2.2, 2.5, 2.6, 2.8, 3.2, 3.3, 3.5, 3.6, 4.1, 4.3.
Non-vaccinated controls: 2.3 and 2.4.
R: right injection site (repeated dose).
L: left injection site (10x overdose).

Table S3B. Histological analysis of the injection sites Study 2 (DB rabbits)

Animal no.	Histological description injection site
1.1 Left	Multifocally perivasculitis in the dermis and subdermal muscular and collagenous tissue, consisting of mononuclear cell aggregation surrounding smaller capillaries and focally a larger arterial blood vessel. Focally small haemorrhage. Multifocally degeneration of muscle fibers. Focally folliculitis with eosinophilic granular cell infiltration.
1.1 Right	Focally minimal degeneration of small single muscle fibers
1.2Left	Mild degeneration of muscle fibers
1.2Right	Focally degeneration of single muscle fibers
1.3Left	Focally minimal mononuclear cell aggregation in the dermis. Few foci of vasculitis with minimal mononuclear cell aggregation surrounding small capillaries. Area with degeneration of single muscle fibers.
1.3Right	Focally de- and regenerative changes in single muscle fibers. Few tiny subdermal hemorrhages. Focally minimal mononuclear cell aggregation in adipose tissue
2.1 Left	Tiny focus of mononuclear cell aggregation in the dermis, small perifollicular hemorrhage
2.1 Right	No abnormalities detected
2.2Left	Focally minimal mononuclear cell aggregation in the dermis
2.2Right	No abnormalities detected
2.3Left	Few foci of vasculitis with minimal mononuclear cell aggregation surrounding small capillaries.Degeneration of single muscle fibers
2.3Right	Multifocally degeneration of muscle fibers. Subcutaneous area with mixed inflammatory cell infiltration (mononuclear cells, macrophages, plasma cells, granulocytes) with karyorrhexis interspersed with degenerative collagen fibers and amorphous irregular coloured material.
2.5Left	Multiple areas with degeneration of muscle fibers.Focally minimal mononuclear cell aggregation surrounding muscle fibers
2.5Right	Minimal degeneration of single muscle fibers
2.6Left	Minimal degeneration of single muscle fibers
2.6Right	Focally degeneration of single muscle fibers
3.1 Left	Multifocally minimal perivascular and/or perineural mononuclear cell aggregation in the dermis. Area with degeneration of muscle fibers
3.1 Right	Dermal small focus of mononuclear cell aggregation. Minimal degeneration of single muscle fibers
4.1 Left	Focally degeneration of single muscle fibers
4.1 Right	Minimal degeneration of single muscle fibers
4.3Left	Minimal degeneration of single muscle fibers
4.3Right	Focally minimal subepidermal mixed inflammatory cell aggregation. Focally minimal degeneration of single muscle fibers.
4.4left	Minimal degeneration of single muscle fibers
4.4Right	Minimal degeneration of single muscle fibers
4.5Left	No abnormalities detected
4.5Right	Minimal degeneration of single muscle fibers

Vaccinates: 1.1; 1.3; 2.2; 2.3; 2.5; 2.6; 3.1; 4.1; 4.3; 4.4 and 4.5
Non-vaccinated controls: 1.2.and 2.1.
Right: right injection site (repeated dose).
Left: left injection site (10x overdose).

Table S4. Summary of antibody titers to RHDV1, RHDV2 and myxoma virus

Table S4A. Antibody titre in SPF rabbits.

	Titre to RHDV1 (log_2)			Titre to RHDV2 (log_2)			Titre to myxoma virus (log_2)		
	**Day 0**	**Day 21**	**Day 35**	**Day 0**	**Day 21**	**Day 35**	**Day 0**	**Day 21**	**Day 35**
**Vaccinates**	<1*	5.3	6.0	<1	6.6	7.5	<6#	14.7	12.5
**Controls**	<1	<1	<1	<1	<1	<1	<6	<6	<6

Table S4B. Antibody titres in DB rabbits.

	Average titre to RHDV1 (log2)			Average titre to RHDV2 (log2)			Average titre to myxoma virus (log2)		
	**Day 0**	**Day 21**	**Day 35**	**Day 0**	**Day 21**	**Day 35**	**Day 0**	**Day 21**	**Day 35**
**Vaccinates**	<1*	4.5	7.7	<1	2.5	3.4	<6#	12.1	13.5
**Controls**	<1	<1	<1	<1	<1	<1	<6	<6	<6

* For RHDV a titre <1 is considered negative.
# For myxoma virus a titre <6 is considered negative.
